# Agronomic, Morphological, and Nutritional Characterization of Greek Traditional Pepper (*Capsicum annuum* L.) Landraces at Commercial and Physiological Maturity for Sustainable and Climate-Smart Vegetable Systems

**DOI:** 10.3390/plants14203164

**Published:** 2025-10-15

**Authors:** Maria Gerakari, Parthenopi Ralli, Anastasia Giannakoula, Georgia Ouzounidou, Antonis Anagnostou, Christos Antoniadis, Ilias D. Avdikos

**Affiliations:** 1 Laboratory of Plant Breeding and Biometry, Agricultural University of Athens, 11855 Athens, Greece; mgerakari@aua.gr; 2 Hellenic Agricultural Organization—DIMITRA (ELGO-DIMITRA), Institute of Plant Breeding and Genetic Resources, 57001 Thermi-Thessaloniki, Greece; pralli@elgo.gr; 3 Laboratory of Plant Physiology and Postharvest Physiology of Fruits, Department of Agriculture, International Hellenic University, Sindos, 57400 Thessaloniki, Greece; agianna@ihu.gr; 4 Hellenic Agricultural Organization-DIMITRA, Institute of Food Technology, 1 S. Venizelou Str., 14123 Lycovrissi, Greece; geouz@elgo.gr (G.O.); antony.anagnostou@gmail.com (A.A.); 5 Laboratory of Vegetable Crop Science, Department of Agriculture, International Hellenic University, Sindos, 57400 Thessaloniki, Greece; ellchris998@gmail.com; 6 Laboratory of Agrobiotechnology and Inspection of Agricultural Products, Department of Agriculture, International Hellenic University, Sindos, 57400 Thessaloniki, Greece

**Keywords:** pepper, organic farming, Greek landraces, nutritional value, yield, climate change

## Abstract

Climate change poses a significant threat to agricultural productivity, particularly in low-input systems where resilient cultivars are crucial. Traditional pepper (*Capsicum annuum* L.) landraces represent a valuable genetic reservoir for adaptation, yet their agronomic and nutritional potential remains underexplored. In this study, twenty-five Greek pepper landraces and commercial varieties were comprehensively evaluated for morphological traits, early and total yield characteristics, and key fruit quality parameters. The results revealed substantial phenotypic diversity in both vegetative and reproductive traits, as well as considerable variation in fruit nutritional composition across the tested genotypes. Notably, ‘Skopos’, ‘Mesoropi’, and ‘Lygaria’ demonstrated superior yield performance, while ‘Pogoniou’, ‘Lyra’, and ‘Kantanou’ excelled in several nutritional quality traits, including high phenolic content, vitamin C, and antioxidant capacity. Heatmap analysis further identified ‘Pogoniou’, ‘Filuria’, ‘Lyra’, ‘Lagada’, and ‘Lygaria’ as consistently ranking among the top performers across yield and quality traits. These findings highlight the dual agronomic and nutritional value of traditional pepper landraces, underscoring their importance as a genetic resource for breeding programs. Overall, the study highlights the importance of conserving and utilizing local pepper germplasm as a sustainable approach to improve productivity, nutritional quality, and resilience in the face of climate change and low-input cultivation systems.

## 1. Introduction

Contemporary lifestyles, along with growing global population pressures, pandemics, and regional conflicts, creates an urgent need for more resilient crop production to ensure global food security [1]. Climate change exacerbates this challenge, already affecting yields through temperature fluctuations, extreme weather events, and disrupted water balances, particularly in arid and semi-arid regions [2]. Moreover, it alters pest and disease dynamics, degrades soil conditions, and affects nutrient availability, making the risk of a food crisis increasingly apparent [3]. From this perspective, numerous studies have explored the impact of climate change on agriculture and highlighted the role of low-input sustainable agriculture in ensuring food security, preserving vital natural resources for human well-being, and contributing to the development of more sustainable agricultural practices [4,5]. Therefore, the effective utilization and optimization of low-input cultivation systems emerge as a key strategy for achieving sustainable food production and enhancing resilience in climate-vulnerable regions, such as the Mediterranean [6,7].

Sweet pepper (*Capsicum annuum* L.) is one of the most popular and highly consumed vegetables worldwide. It originated in Mexico and Central America regions and spread globally, comprising the world’s most important type of spice, which provides nutritional value to consumers, particularly vitamin A and E, as well as flavoring and coloring food [8]. Sweet peppers are generally recognized as a potential food source of vitamins, phenolic compounds, carotenoids, and flavonoids, which have been shown to possess known positive health effects [9,10]. The high diversity of its fruit shapes and colors, is often related to its maturation degree, and its pungency, specific taste and/or distinct aroma make sweet peppers very popular and an excellent ingredient to be included in many types of diets and dishes with high attractiveness for several types of consumers [11,12].

Given the growing environmental challenges posed by climate change, the adoption of sustainable agricultural practices—particularly in organic fruit and vegetable production—has increased significantly. In this context, *Capsicum* landraces and traditional varieties, which are well adapted to local environmental conditions, represent a valuable reservoir of genetic material. These peppers are remarkable for their rich nutritional profile, especially their high phenolic and flavonoid content, which contributes to their added value. Over the last few decades, flavor has been a focus in breeding programs due to consumers’ continuous complaints about the loss of traditional organoleptic characteristics in vegetables [13,14]. Furthermore, the broad genetic diversity found in local varieties enhances their potential for adaptation to organic farming systems and enables the effective exploitation of genotype × environment interactions to select genotypes with high nutritional value and at the same time well adapted to challenging environments [15,16].

**Figure 1:**
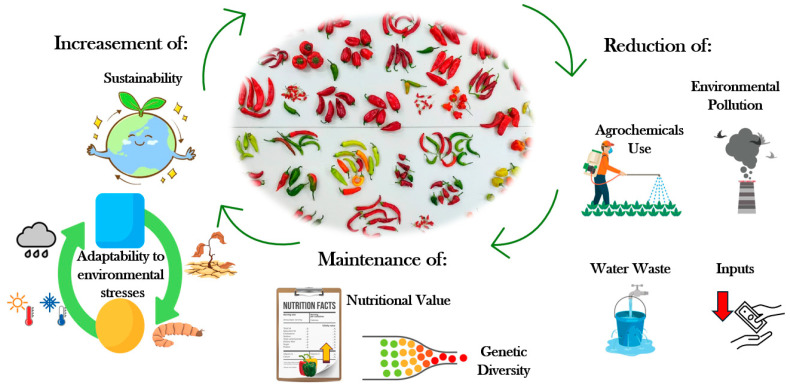
Advantages of pepper landrace exploitation under low-input cultivation systems.

The present study evaluates twenty-five pepper genotypes, including nineteen landraces from the collection of the Greek Genebank (ELGO-DIMITRA), one conservation variety and five commercial varieties derived from local populations, under a low-input cultivation system, with a focus on evaluating physiological characteristics, yield performance, and fruit nutritional value. Given the high production costs and the potential yield limitations currently challenging agriculture globally, particularly in Greece, including pepper cultivation, identifying resilient genotypes that can thrive under low-input conditions is of critical importance. Traditional varieties, often better adapted to local agro-environmental conditions, represent a valuable reservoir of genetic diversity that can support more sustainable and climate-resilient production systems. Their preservation and utilization not only enhance agrobiodiversity but also contribute to the promotion of locally adapted, high-quality produce with added economic and gastronomic value (Figure 1).

## 2. Results

### 2.1. Descriptive Characteristics

Descriptive characteristics provide a valuable source of information regarding the phenotypically expressed traits of a genotype. In the present study, several important descriptive characteristics were evaluated, providing information regarding key morphological characteristics for each pepper genotype tested, including plant height, stem length, leaf length, and leaf width (Table 1). These characteristics offer insights concerning the architectural diversity and potential adaptability of the genotypes under low-input cultivation system. The highest plants were recorded in ‘Lyra’ (37.33 cm) and ‘Platika Florinis’ (37.00 cm), which also exhibited long stems, indicating vigorous vegetative growth. In contrast, ‘Lagada’ and ‘Akamatra’ presented compact growth, demonstrating the shortest plants (14.67 cm and 16.33 cm, respectively) and the shortest stem lengths, which might be a valuable trait for high-density cultivation. Leaf measurements varied notably among the tested genotypes. Genotypes like ‘Lyttos’ and ‘Galatista’ exhibited the widest leaves (4.68 cm and 4.67 cm, respectively), a trait that may potentially lead to increased photosynthetic capacity. On the contrary, ‘Filyria’ exhibited the narrowest leaves (2.52 cm), which may reflect a more determinate or drought-tolerant genotype (Table 1).

Information regarding the fruits’ morphological characteristics, including fruit length, diameter, the ratio of length to diameter as well as the fruit shape in longitudinal section are presented in Table 2. More specifically, ‘Mesoropi’ genotype exhibited the longest and most slender fruits, with the highest length/diameter ratio (10.50), classifying it clearly as elongated and bullet-shaped, according to UPOV. Similarly, in ‘Ierapetras’ and ‘Anarahi’ genotypes high elongation indices (9.67 and 8.71, respectively) were observed, reinforcing their classification as elongated or tapered fruit types. Contrary, the pepper genotypes ‘Lyttos’, ‘Akamatra’, and ‘Galatista’ produced rounder fruits, with length/diameter ratios close to 1.63 or less. Particularly, ‘Galatista’ presented a notably low ratio (1.27), consistent with its classification as oblate or nearly spherical. Moreover, ‘Lygaria’ genotype displayed a large fruit diameter (3.41 cm) with moderate length (11.05 cm), suggesting a more blocky or rectangular shape. ‘Apollonas’ also had the largest diameter (5.04 cm) but short length (5.56 cm), indicating a squat or flattened morphology. As a general outcome, fruit shape in longitudinal section classification revealed that most of the tested traditional pepper genotypes were classified between 5 and 7 (rectangular to triangular), with ‘Lagada’ landrace presenting an impressive score of 9, reflecting a unique and extreme morphology linked to a specialized genetic background (Table 2).

Internal fruit characteristics were measured in the present research, including the number of locules, pericarp (flesh) thickness, and capsaicin presence in the placenta—attributes that are crucial for determining fruit structure, culinary applications, and pungency (Table 3). More specifically, noteworthy was the fact that the number of locules varied widely among the studied genotypes, with the majority exhibiting single-locule fruits (code 1.0), such as ‘Kantanou’, ‘Anarahi’, and ‘Evriaki’. Genotypes like ‘Lygaria’, ‘Lyttos’, and ‘Akamatra’ had multi-locular fruits (code 5.0), potentially enhancing seed number and internal volume. Moreover, pericarp thickness ranged from 1.0 mm (Evriaki’, ‘Lyra’, ‘Filyria’, ‘Mesoropi’) to 9.0 mm (‘Lygaria’, ‘Apollonas’), the latter suggesting suitability for fresh market types requiring thick-walled, firm fruits. Intermediate values (e.g., 5–7 mm) were observed in genotypes such as ‘Kantanou’ and ‘Sykousis’, balancing firmness with processing flexibility. Regarding the presence of capsaicin in the placenta (as a marker for pungency), the results showed that eight out of twenty-five samples, such as ‘Pogoniou’, ‘Anarahi’, ‘Lyra’, and ‘Evriaki’, demonstrated this presence (9.0).

### 2.2. Yield Characteristics

Accurate data on both early and total yield are essential in plant experiments, as they provide critical insights into crop performance, growth dynamics, and the potential of genotypes to sustain productivity under diverse environmental conditions. Table 4 presents the performance of the twenty-five pepper genotypes in terms of early yield components, including the number of early fruits per plant, early yield (g per plant), and average early fruit weight (g). Significant variability was observed among the tested genotypes. Notably, ‘Mesoropi’ exhibited the highest early plant yield (163.40 g) and the highest number of early fruits (10.80), indicating a high early productivity potential. In contrast, ‘Galatista’ recorded no early production (0.00), while ‘Lygaria’ showed the highest average early fruit weight (46.51 g), despite having a relatively low number of fruits (2.57). Among the landraces, ‘Filyria’ and ‘Akamatra’ demonstrated high early fruit numbers (9.33 and 9.03, respectively), though coupled with lower fruit weights (9.60 g and 1.66 g), suggesting a higher sink number but smaller individual fruit size. On the other hand, ‘Kentavros’ and ‘Skopos’ balanced moderate fruit numbers with high fruit weights and yields.

Table 5 summarizes the total number of fruits per plant, the total yield (g per plant), and the average fruit weight (g) across the tested genotypes. The results demonstrate a broad-spectrum regarding yield potential and fruit size among the genotypes. The highest total yield was recorded in ‘Skopos’ (627.74 g), accompanied by a relatively high fruit number (15.50) and large average fruit weight (39.01 g), indicating excellent performance in cumulative productivity. ‘Mesoropi’ and ‘Lygaria’ also showed high yields (455.57 g and 401.93 g, respectively), albeit with differing strategies. In ‘Mesoropi’, the highest fruit number was observed (36.12), while ‘Lygaria’ had fewer fruits but heavier ones (34.87 g). Genotypes like ‘Filyria’ and ‘Lyra’ produced a large number of small-sized fruits, reflecting a yield strategy based on fruit count rather than size. On the contrary, genotypes like ‘P14’ and ‘Apollonas’ combined moderate fruit numbers with high fruit weights (41.62 g and 33.59 g, respectively), contributing to elevated yields. Genotypes like ‘Akamatra’ and ‘Lagada’ exhibited extremely low total yields (15.38 g and 41.11 g, respectively).

### 2.3. Fruit Quality and Nutritional Value Characteristics

Evaluating the quality and nutritional value characteristics of fruit in traditional pepper landraces and commercial varieties is crucial, as these traits determine not only consumer acceptance and marketability but also the nutritional contribution and potential use of traditional germplasm in breeding programs. The present study records data on the instrumental color parameters of green fruits harvested at commercial maturity, including lightness (L*), the red-green coordinate (a*), and the yellow-blue coordinate (b*) (CIELAB). These parameters reflect both the physiological status of the fruit at the harvest stage and its visual appeal, which is a crucial market-accepted trait. Furthermore, the L* values, which express brightness, were observed to range from 38.96 (‘Bachovitiki’) to 68.34 (‘Anarahi’), indicating high variability in fruit lightness. Genotypes like ‘Anarahi’, ‘Lagada’, and ‘P13’ exhibited the highest L* values (>66), suggesting brighter green hues potentially linked to thinner cuticles or lower chlorophyll density. The a* values (green to red axis) ranged from 6.08 (‘Evriaki’) to 23.82 (‘Filyria’). Higher values were recorded in genotypes such as ‘Filyria’, ‘Pogoniou’, and ‘Skopos’, demonstrating a greater tendency toward early red pigmentation or chlorophyll breakdown, even at the commercial maturity stage. Concluding, as for the b* coordinate (blue to yellow axis), the highest values were found in ‘Ano Gavrio’ and ‘Galatista’ (>49), denoting more yellowish tones. Lower b* values were observed in ‘Evriaki’ and ‘Bachovitiki’, indicating greener or duller hues (Table 6).

Mechanical firmness (texture) and biochemical parameters—total phenolic content (expressed as gallic acid equivalents) and vitamin C concentration—in green fruits harvested at commercial maturity are presented in Table 7. Significant variation was observed among the pepper genotypes in terms of fruit firmness. The highest values were recorded in ‘Apollonas’ (14.90 N) and ‘Ano Gavrio’ (13.50 N), indicating denser and mechanically resistant fruits. In contrast, ‘Anarahi’, ‘Arnaouti’, and ‘Ierapetras’ showed the lowest firmness (<6 N), suggesting softer textures more susceptible to postharvest damage. Total phenolic content ranged widely, from 24.34 mg/100 g FW (‘Florinis’) to 137.80 mg/100 g FW (‘Lagada’) and 134.00 mg/100 g FW (‘Pogoniou’), reflecting both genetic and physiological differences. Genotypes like ‘Filyria’ and ‘Lyra’ also demonstrated elevated polyphenol levels, which are often linked to antioxidant capacity and potential nutraceutical value. Moreover, vitamin C content was presented as highest in ‘Lygaria’ (205.00 mg/100 g FW), followed by ‘Filyria’ (143.00 mg) and ‘Pogoniou’ (128.10 mg), all substantially exceeding typical values reported in pepper germplasm. In contrast, ‘Akamatra’, ‘Kentavros’, and ‘Mesoropi’ exhibited the lowest concentrations (<20 mg), suggesting limited nutritional density (Table 7).

The biochemical profiling parameters of green fruits harvested at commercial maturity, including antioxidant capacity (FRAP, μg/g FW), flavonoids (mg/100 g FW), and porphyrins (mg/g FW) have also been evaluated (Table 8). These compounds are vital indicators of nutritional quality and oxidative stress tolerance. In terms of characteristics, FRAP, flavonoids, and porphyrins showed no statistically significant differences between the pepper genotypes. Regarding FRAP measurements, which is indicative of the fruit’s antioxidant capacity, high values were observed for the most genotypes (often >600 μg/g FW), with ‘Filyria’ (1079.50 μg/g FW) and ‘Lagada’ (968.23), outperforming. Even genotypes with moderate phenolic content exhibited strong FRAP values, implying that other compounds (e.g., ascorbic acid, flavonoids) contribute significantly to antioxidant capacity. Moreover, flavonoid content ranged from 9.30 (‘Galatista’) to 27.02 mg/100 g FW (‘Lagada’), with ‘Lyra’, ‘Filyria’, and ‘Pogoniou’ also exceeding 24 mg. These results indicate notable inter-genotypic differences in secondary metabolism that can be leveraged in breeding for functional food traits. Regarding porphyrin content, although relatively consistent among genotypes, it reached maximum levels in ‘Pogoniou’ (0.83 mg/g) and ‘Filyria’ (0.82 mg/g). The ‘Apollonas’ genotype exhibited the lowest value (0.23 mg/g) (Table 8). These compounds serve as precursors in chlorophyll and heme biosynthesis, and may influence plant stress responses.

The color attributes of pepper fruits at the stage of physiological maturity, expressed in the CIELAB color space: L* (lightness), a* (red-green component), and b* (yellow-blue component) have also been determined (Table 9). These parameters are indicative of ripeness, pigment accumulation, and potential market value. L* values ranged from 34.34 (‘Lyra’) to 47.30 (‘Ano Gavrio’), reflecting significant variation in fruit brightness among genotypes. Lighter red fruits were observed in ‘Ano Gavrio’ and ‘Akamatra’, whereas darker red fruits characterized genotypes like ‘Lyra’ and ‘Pogoniou’. The a* values, representing the red color intensity, were consistently high across genotypes, reflecting substantial accumulation of carotenoid pigments. The highest a* values were recorded in ‘Lagada’ (41.40), ‘Mesoropi’ (41.14), ‘Arnaouti’ (40.77) and Ierapetras (40.74), which are associated with a deeper red hue. In peppers, this redness is primarily attributed to capsanthin, the dominant red carotenoid in Capsicum species, along with capsorubin and, to a lesser extent, lycopene. As for the b* values, which describe the yellow-orange component, they exhibited broader variability—from 17.15 (‘Skopos’) to 46.17 (‘Akamatra’). Elevated b* values may be indicative of higher concentrations of yellow-orange carotenoids such as β-carotene, zeaxanthin, and lutein. Genotypes like ‘Akamatra’, ‘Ano Gavrio’, and ‘Galatista’ demonstrated the highest b* values (>33), suggesting potential for enhanced carotenoid diversity and nutritional value (Table 9).

Key postharvest quality attributes of red pepper fruits at physiological maturity—such as firmness, total phenolic content (gallic acid equivalents), and vitamin C concentration (mg/100 g FW)—were also estimated in the present research, as these parameters are critical indicators of fruit shelf life, nutritional value, and overall consumer quality (Table 10). More specifically, fruit firmness ranged from 1.79 N (‘Platika Florinis’) to 15.50 N (‘Lagada’), with genotypes like ‘Lagada’, ‘Apollonas’, and ‘Lyra’ demonstrating superior resistance to deformation. These traditional varieties could serve as ideal candidates for extended shelf-life and transportation. Conversely, ‘Filyria’, ‘Arnaouti’, and ‘Lygaria’ exhibited the lowest firmness (<6 N), indicating greater susceptibility to bruising and perishability. Regarding total phenolic content values, they varied from 61.42 mg/100 g FW (‘Mesoropi’) to 165.47 mg/100 g FW (‘Skopos’), including other highly ranked genotypes like ‘Pogoniou’, ‘Filyria’, and ‘Lyttos’ (Table 10). These genotypes may be considered as elite sources of phenolic antioxidants in pepper germplasm. Moreover, vitamin C content demonstrated notable variation, from 24.42 mg/100 g FW (‘Bachovitiki’) to 167.59 mg/100 g FW (‘Pogoniou’). Other genotypes with a high-vitamin profile were ‘Skopos’, ‘Filyria’, and ‘Evriaki’, which presented increased nutritional appeal. In contrast, ‘Florinis’, ‘Apollonas’, and ‘Ano Gavrio’ demonstrated lower ascorbic acid concentrations (<60 mg), indicating more limited antioxidant capacity (Table 10).

Table 11 provides information on key biochemical parameters of red pepper fruits, harvested at the physiological maturity stage, including total phenolics, antioxidant capacity (FRAP), flavonoid content, and porphyrin concentration. FRAP values ranged from 251.29 (‘Ano Gavrio’) to 1152.13 μg/g FW (‘Lyttos’), showing wide differences in total antioxidant capacity. Notably, ‘Anarahi’ and ‘Kantanou’ also displayed high FRAP values, suggesting their potential to counteract oxidative stress through multiple mechanisms. Regarding flavonoid content, it was observed to be highest in ‘Kantanou’ (28.63 mg/100 g FW), Lyra (27.17 mg) and ‘Votsi’ (27.05 mg), with ‘Lagada’, ‘Anarahi’, ‘Lygaria’, and ‘P13’ also showing high levels (>25 mg). These compounds contribute not only to antioxidant capacity but also to fruit color and potentially increased nutritional value characteristics. Furthermore, porphyrin levels, although more narrowly distributed, peaked in ‘Kantanou’ and ‘P13’ (both 0.90 mg/g), indicating a potential role in fruit metabolism and photodynamic response. ‘Kentavros’ and ‘Florinis’ exhibited the lowest values (<0.60 mg/g) regarding the porphyrin levels.

### 2.4. Hierarchical Cluster Analysis and Ranking

**Figure 2:**
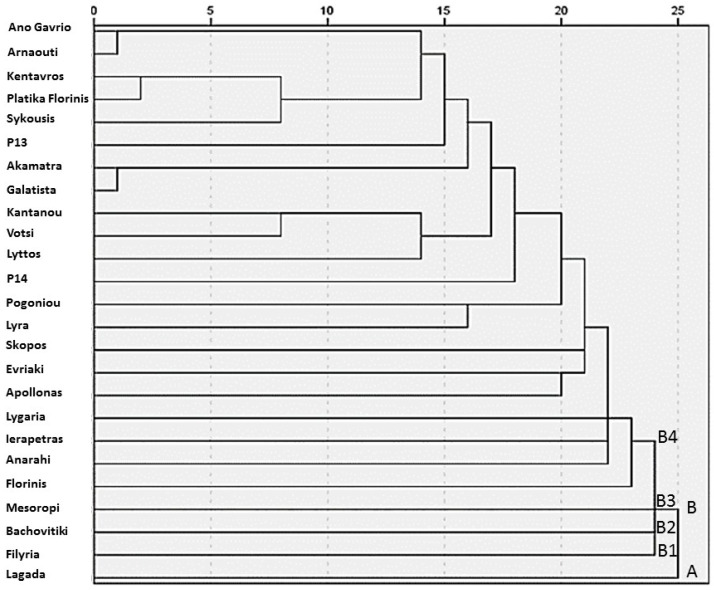
Dendrogram of 25 pepper (*Capsicum annuum* L.) genotypes generated using hierarchical cluster analysis (average linkage, between groups) based on combined morphological, agronomic, and nutritional traits. The vertical axis represents the rescaled distance cluster, while the horizontal axis indicates the genotype numbers as listed in all the tables above. Letters A and B (B1, B2, B3, & B4) indicate the main clusters and sub-clusters identified in the dendrogram.

The hierarchical cluster analysis conducted generated a dendrogram that revealed distinct grouping patterns among the 25 traditional pepper (*Capsicum annuum* L.) genotypes (Figure 2). Based on the rescaled distance cluster combine values, two primary clusters, A and B, were identified, reflecting the considerable phenotypic variability present within the studied germplasm. Cluster A consisted of a single landrace, ‘Lagada’, which was clearly differentiated from all others, likely due to its unique morphological or nutritional characteristics. Cluster B encompassed the remaining 24 genotypes and was further subdivided into four sub-clusters: sub-cluster B1 consisted solely of ‘Filyria’, sub-cluster B2 included only ‘Bachovitiki’, sub-cluster B3 was represented by ‘Mesoropi’ and sub-cluster B4 included all other genotypes grouped under this broader cluster. Despite the overall phenotypic diversity, the dendrogram also highlighted several pairs of genotypes that clustered at very short linkage distances, indicating high levels of similarity or possible genetic relatedness. Specifically, ‘Ano Gavrio’ and ‘Arnaouti’ appeared nearly indistinguishable, suggesting strong morphological or agronomic resemblance, which may be attributed to their common insular origin, as both landraces derive from Aegean islands. Similarly, ‘Kentavros’ and ‘Platika Florinis’ formed a closely related pair, as did ‘Akamatra’ and ‘Galatista’, potentially reflecting common ancestry, geographic proximity, or parallel farmer selection histories. These clustering patterns underscore the rich phenotypic structure within the collection and may inform targeted conservation strategies or the rational selection of parental lines for breeding programs aimed at enhancing pepper adaptation and fruit quality under diverse environmental conditions.

**Figure 3:**
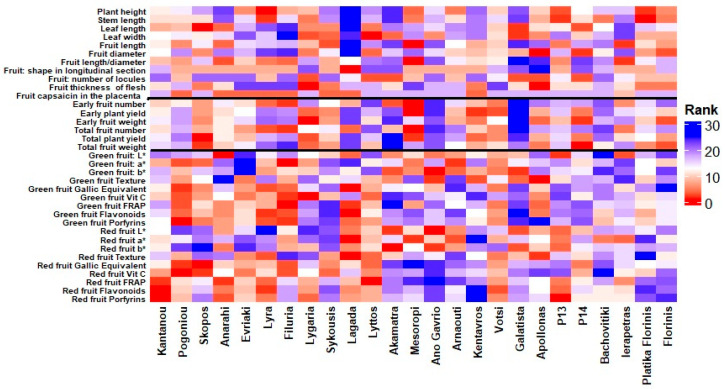
Heatmap showing the ranking of 25 pepper (*Capsicum annuum* L.) entries across evaluated traits. Warmer colors (red) indicate higher rankings, while cooler colors (blue) represent lower rankings. Vertical black lines separate the three main trait groups: morphological characteristics (left), yield-related traits (middle), and fruit quality and nutritional value traits (right).

The heatmap in Figure 3 illustrates the ranking patterns of the 25 pepper entries across a comprehensive set of evaluated traits. The vertical black lines divide the traits into three main categories: morphological characteristics (left section), yield-related traits (middle section), and fruit quality and nutritional value traits (right section). Warmer colors (red shades) indicate higher ranking performance for the respective traits, whereas cooler colors (blue shades) indicate lower rankings. The visualization highlights the considerable variation among entries, with some genotypes, such as ‘Pogoniou’, ‘Filuria’ and ‘Lagada’, consistently ranking high in several fruit quality and nutritional parameters, while others, like ‘Mesoropi’, ‘Lygaria’, and ‘Apollonas’ excel in certain yield-related or morphological traits. This pattern highlights the diverse performance profiles of the evaluated germplasm and the potential for targeted selection based on specific breeding objectives. However, suppose we aim to estimate the overall ranking of the tested genotypes. In that case, the heatmap analysis indicates that the landraces ‘Pogoniou’, ‘Filuria’, ‘Lyra’, ‘Lagada’, and ‘Lygaria’, consistently ranked among the top performers across all the measured traits, highlighting their potential as promising candidates for breeding and low-input cultivation systems.

## 3. Discussion

Climate change has been recognized as a significant challenge for global agriculture, but its impact has intensified to unprecedented levels in recent years. Rising temperatures, erratic rainfall patterns, prolonged droughts, and increasing soil and water salinity are no longer future projections but present-day realities that severely threaten crop productivity and food security [17]. What was once a broad topic of discussion has now become an urgent crisis, demanding immediate action. More than ever, the agricultural sector must adopt sustainable and efficient solutions that ensure both environmental resilience and long-term productivity [18]. Addressing this challenge requires an integrated approach that combines innovative agronomic practices, the use of resilient genotypes, the maintenance of plant species genetic diversity, and the optimization of resource use to safeguard farming systems in the face of escalating climate pressures [19].

Pepper landraces represent a valuable genetic resource for enhancing resilience to climate change, offering adaptive potential and stable yields under low-input cultivation systems [20]. Characterizing the genetic diversity within the *Capsicum* genus is crucial for pepper breeding programs, given its global significance as both a vegetable and spice crop. Exploring this rich genetic diversity traditional varieties with unique and valuable traits, can be uncovered [21].

In a recent study, 17 pepper landraces were evaluated for 20 qualitative and 45 quantitative traits, including plant height, stem and leaf dimensions, fruit morphology, and pericarp thickness, leading to the conclusion of increased variability among the selected traditional pepper landraces [22]. The research pointed out that phenotypic characterization serves as a valuable approach to complement nutritional and genetic analyses in crop diversity studies, helping to identify promising accessions for use in breeding programs. Tripodi and Greco also conducted a similar comprehensive study in 2018, in which a large-scale phenotypic characterization of 307 *Capsicum* accessions from 48 regions and 9 species was performed using both conventional descriptors and semi-automated high-throughput tools to evaluate plant, leaf, flower, and fruit traits. The study’s results indicated significant morphological variation among accessions and species, highlighting extensive diversity and suggesting that domestication and selection had increased variability, particularly in fruit shape and color [23]. Similar results are also demonstrated in our study, where the comprehensive evaluation of the 25 pepper genotypes revealed substantial phenotypic diversity in both vegetative and reproductive traits, highlighting their potential for selection and breeding. More specifically, in our experiment, the evaluated morphological traits—such as plant height, stem length, and leaf dimensions—demonstrated significant variation. Genotypes like ‘Lyra’, ‘Platika Florinis’, and ‘P13’ were among the tallest and most vigorous, indicating potential for high biomass production. In contrast, ‘Lagada’ and ‘Akamatra’ exhibited compact growth, which may be suitable for dense planting or pot cultivation. Leaf size was highest in ‘Lyttos’ and ‘Galatista’, suggesting greater photosynthetic potential. In terms of fruit morphology, clear differences were noted in shape, size, and length-to-diameter ratios among the examined genotypes. Consistent with previous findings of the existing literature, our study further supports the extensive phenotypic variability among traditional pepper genotypes, highlighting their value as genetic resources for breeding and adaptation, especially under low-input cultivation systems, where their performance was thoroughly assessed.

Moreover, a study regarding the genetic diversity of the fruit in 168 pepper accessions, collected from 62 locations across six different Balkan countries, revealed significant variation in shape, size, and color of the fruit. The pepper accessions were grouped into distinct morphological types and clustered into eight groups based on fruit traits. The analysis demonstrated that fruit size, end shape, and color were major contributors to diversity. The findings highlight the rich, yet threatened, diversity of Balkan peppers and provide valuable insights for breeding programs and future genetic studies on fruit morphology [24]. In a similar experiment carried out in the same geographic area, 15 Serbian pepper genotypes (including 11 populations and 4 varieties derived from local landraces) were evaluated based on morphological traits, such as fruit weight, length, width, pericarp thickness, shape, and number of chambers, as well as phytochemical parameters. The results presented one population with high internal diversity, suggesting it as suitable for individual selection, and three populations that were identified as promising candidates for use in recombination breeding programs aimed at improving desirable traits [25]. The findings of our study align with the above research results, which reveal high variability in the fruit characteristics of the 25 tested genotypes. More specifically, ‘Mesoropi’ and ‘Ierapetras’ had the most elongated fruits, while ‘Lyttos’ and ‘Apollonas’ produced wider or more rounded fruits. Genotypes such as ‘Lygaria’ and ‘Apollonas’ also had thicker fruit flesh, an important trait for quality and marketability. Shapes ranged from rectangular and heart-shaped to bullet-shaped, offering superior variability and numerous options for different culinary or industrial uses.

Earliness is a highly desirable trait for pepper growers, particularly under stress-prone environments and short cultivation cycles. In our experiment, for the early yield measured characteristics, the early fruit number varies from 0.00 (‘Galatista’) to 10.80 (‘Mesoropi’), presenting variation among the tested genotypes. Early plant yield was also led by ‘Mesoropi’ (163.40 g), followed by ‘Kentavros’ (142.30 g), ‘Votsi’ (122.10), and ‘Lygaria’ (120.10), while the commercial variety ‘P14’ (115.90 g) ranked only fourth. Early fruit weight is presented more increased in ‘Lygaria’ (46.51 g) followed by ‘P14’ (42.63 g), ‘Florinis’ (41.62 g) and ‘Apollonas’ (40.16 g).

Regarding the measured characteristics of the total yield, several interesting findings are noted. ‘Mesoropi’ is consistently first regarding total fruit number (36.12) and among the first for plant yield (455.57 g), maintaining the same good performance for these two traits as in early yield and a medium total fruit weight value (13.96 g). ‘Skopos’ outperforms in terms of total plant yield (627.74 g), while it is the second-best landrace in terms of total fruit weight (39.01 g). However, genotypes ‘Sykousis’, ‘Kentavros’, ‘Votsi’ and ‘P14’ did not excel in total plant yield, following their early plant yield performance. Additionally, the commercial variety ‘P14’ genotype demonstrated the highest fruit weight in total yield (41.62 g) compared to the other 24 genotypes. The results above demonstrate that early and total yield measurements do not always align, making it difficult to accurately predict the total yield based only on early yield data. Each genotype may exhibit different performance at each developmental stage, likely due to various unknown genetic factors that control gene expression, resulting in unpredictable final yields. However, several genotypes, such as ‘Mesoropi’, appear to be consistent and reliable in their performance across both yield stages. This is particularly important considering that the evaluation was carried out under low-input and stress conditions. Under such conditions, several non-improved traditional landraces either matched or even exceeded the productivity of an improved Greek variety (‘P14’) and a conservation variety (‘Bachovitiki’), both registered in the national catalogue.

Several studies have reported that total fruit yield is positively associated with fruit number per plant, pericarp thickness, and fruit diameter, making these traits important selection criteria for yield improvement [26,27]. However, this association was not clearly observed in the present study, where the highest total yields were recorded for ‘Skopos’ (627.74 g), ‘Mesoropi’ (455.57 g), and ‘’Filyria’ (401.93 g), with only ‘Mesoropi’ also displaying a high total fruit number. The correlation coefficient (Pearson) between total yield and fruit number was statistically significant but relatively low (ρ_p_ = 0.48). In a closely related species, like tomato, this relationship is typically reported as very strong and highly significant. For example, Avdikos et al. in a research conducted in 2021 [14] reported a correlation coefficient of ρ_P_ = 0.97 between yield per plant and fruit number per plant in tomatoes. In contrast, the three landraces with the highest pericarp thickness (7 mm, 11 mm, and 9 mm, respectively) did not correspond to the highest-yielding ones, and the correlation between total yield and pericarp thickness was not statistically significant (ρ_P_ = 0.21). Similarly, not all high-yielding landraces exhibited large fruit diameters (3.21 cm, 1.52 cm, and 3.41 cm, respectively), and the correlation with total yield was also weak (ρ_P_ = 2.78).

In general, the 25 tested genotypes displayed considerable variability in their morphological and yield-related traits, reflecting the rich genetic diversity within traditional germplasm. Certain genotypes, such as ‘Mesoropi’, ‘P14’, ‘Skopos’ and ‘Filyria’, combined strong performance in both early and total yield, making them promising candidates for breeding programs targeting overall productivity. On the other hand, varieties like ‘Kentavros’, ‘Votsi’, and ‘Sykousis’ may be more suitable for early harvests with smaller-sized fruits, offering alternative options for growers aiming to capture early market advantages. This variability underlines the fact that not all growers share the same priorities. Some may emphasize early production to secure higher market prices, while others may prioritize overall yield or fruit size for specific markets and processing purposes. The observed diversity among the genotypes, therefore, should not be viewed as a limitation but rather as a valuable reservoir of traits that can serve different cultivation goals and management practices.

The evaluation of Greek pepper landraces of the present study revealed substantial variability in fruit quality and nutritional value traits, particularly in phenolics, vitamin C, and antioxidant capacity (FRAP), aligning with findings reported for Tunisian hot pepper landraces [28]. In both studies, phenolic content emerged as a key discriminating factor, with genotypes such as ‘Skopos’, ‘Filyria’ and ‘Lyttos’ in our work, and ‘Cayenne’ in the Tunisian collection, exhibiting superior levels. Similarly, the high antioxidant potential, as characterized by the FRAP in our dataset, parallels the elevated radical scavenging activity recorded for ‘Cayenne’ in both hydrophilic and lipophilic fractions [28] and in our work in ‘Kantanou’, ‘Anarahi’, and ‘Lyttos’. While the Tunisian landraces showed relatively low genetic variability in vitamin C, our results demonstrated a broader range. These converging observations across distinct agro-climatic regions reinforce the potential of local pepper germplasm as a valuable reservoir of bioactive-rich, high-quality material.

In another recent study, the assessment of 23 *Capsicum annuum* varieties was evaluated, integrating morphological, agronomic, and nutritional analyses to guide breeding and genetic resource management [29]. In accordance with our findings, this study also revealed substantial variability in ascorbic acid content; however, our results covered a broader range than the 125.2–331.4 µg g^−1^ FW reported in their work, highlighting genotypes such as ‘Skopos’, ‘Filyria’, and ‘Pogoniou’ for their high vitamin C levels. In both datasets, significant differences were observed among varieties for bioactive compounds, with phenolics and antioxidant capacity emerging as important discriminating traits in our case, while their study also emphasized variation in soluble protein, sugar, and organic acid contents.

Across the 25 Greek pepper varieties, cross-stage rank concordance (green vs. red) for quality and nutritional traits was generally weak: texture (Spearman’s ρ_S_ = 0.35), total phenolics expressed as gallic acid equivalents, GAE (ρ_S_ = 0.29), phenolics (ρ_S_ = 0.37), FRAP (ρ_S_ = 0.25), flavonoids (ρ_S_ = 0.22), and porphyrins (ρ_S_ = 0.23), whereas vitamin C showed strong concordance (ρ_S_ = 0.62, *p* < 0.01). These results indicate substantial genotype-by-stage re-ranking: landraces that rank high for nutritional attributes at commercial (green) maturity do not necessarily retain their advantage at physiological (red) maturity. Practically, growers and breeders should match selection and harvest decisions to the target stage—e.g., choose genotypes with stable rank profiles when flexible harvest windows are desired, or stage-specific “winners” when aiming to maximize a particular compound at a given maturity. Notably, vitamin C exhibited strong cross-stage stability, while antioxidant-related metrics (TPC/GAE, FRAP, flavonoids) were more stage-dependent, suggesting that selection for nutritional quality may need to be stage-specific rather than inferred from a single harvest stage.

Several studies have highlighted the remarkable variability of bioactive compounds among pepper genotypes and emphasized the potential of landraces and other *Capsicum* species such as *C. chinense* and *C. baccatum* to be considered functional foods [28,30]. Our results clearly demonstrate that Greek traditional varieties, both at the green (commercial maturity) and red (physiological maturity) stage, exhibit nutritional values that are equal to or surpass those reported in the international literature. For instance, the ascorbic acid content of the landraces ‘Lygaria’ (205 mg/100 g FW) and ‘Filyria’ (143 mg/100 g FW) in green fruits exceeded the ranges described by Howard et al. [31], who reported 63–172 mg/100 g FW in immature fruits of sweet peppers depending on cultivar. Similarly, ‘Pogoniou’ and ‘Lagada’ displayed exceptionally high total phenolic content in green fruits (>130 mg/100 g FW), values that were more than double those reported in most studies, such as in Howard’s et al. [31] (13–58 mg/100 g FW), Loizzo’s et al. [32] (~30–120 mg/100 g FW), and Martínez et al. [22] (~30–120 mg/100 g FW). These findings highlight the outstanding antioxidant potential of Greek landraces and confirm the strong genotype effect on the nutritional profile of peppers.

It is generally accepted that ripening enhances the accumulation of carotenoids, phenolic compounds, and antioxidant activity, although the magnitude of these changes depends on both genotype and growing environment [31,33,34]. In our study, a clear increase was observed from commercial to physiological maturity, with mean increments of 33% in total phenolics, 73% in vitamin C, 39% in antioxidant capacity, 31% in flavonoids, and 34% in porphyrins. To contextualize the mature-stage values, previous studies have reported vitamin C concentrations in ripe peppers of 74.6–202.4 mg/100 g FW [31], ~130 mg/100 g FW [35], and 132–200 mg/100 g FW [28] in our material, red fruits reached 168 mg/100 g FW (‘Pogoniou’), which falls within these published ranges.

Total phenolic content and antioxidant activity in red-ripe peppers have been consistently reported among the highest levels observed in vegetables. Ghasemnezhad et al. in 2011 [35] reported total phenolics up to ~120 mg/100 g FW in *C. annuum* varieties, while Alghamdi et al. in 2025 [36] reported 160 mg/100 g FW, and Chouikhi et al. in 2024 [28] recorded 30.3 mg/100 g FW. Comparable or even superior values were observed in our material, with the landrace ‘Skopos’ reaching 165.5 mg/100 g FW.

In terms of antioxidant activity, the highest FRAP values in our study were recorded in the landraces ‘Kantanou’, ‘Anarahi’, and ‘Lyttos’, exceeding 1100 μg/g FW. Comparatively, Constantino et al. in 2020 [37] reported much lower values (52.6–568.2 μg/g FW) across 22 Brazilian pepper accessions. This highlights the considerably higher antioxidant potential of Greek landraces, further reinforcing their value as reservoirs of nutritional quality traits and as promising candidates for the development of functional foods.

Taken together, our comparative analysis strongly supports the concept that several Greek pepper landraces (e.g., ‘Pogoniou’, ‘Lyra’, ‘Kantanou’, and ‘Filyria’) are not only competitive at the international level but also represent valuable germplasm resources that could be marketed and promoted as functional foods due to their superior bioactive profile.

Currently, there is an evident shortage of cultivars bred explicitly for the particular requirements of sustainable agricultural systems. In developed countries, it is estimated that more than 95% of organic agriculture still relies on cultivars initially developed for conventional high-input systems, which generally lack the traits required to perform well under low-input or stress-prone environments [38]. In light of climate change and the urgent need to identify resilient and productive varieties capable of thriving under extreme environmental conditions, alongside the European Green Deal targets that call for the development of organic cultivars tailored to sustainable farming contexts, traditional landraces emerge as the most valuable reservoir. The comprehensive evaluation of Greek pepper landraces conducted in this study demonstrates their high potential as breeding material, particularly through the combination of yield-stable genotypes such as ‘Skopos’, ‘Mesoropi’, and ‘Lygaria’ with nutritionally superior landraces like ‘Pogoniou’, ‘Lyra’, and ‘Kantanou’, which exhibited elevated phenolic content, vitamin C levels, and antioxidant capacity—traits of direct relevance for breeding programs aiming to improve both productivity and fruit nutritional quality. Greece, renowned for its exceptional biodiversity across multiple vegetable species, with pepper landraces serving as a prime example, can act as a model for the conservation and utilization of traditional germplasm. Harnessing this diversity through targeted breeding strategies represents a crucial pathway for developing next-generation cultivars that are adapted to the challenges of climate change and the demands of low-input and organic agriculture.

## 4. Materials and Methods

### 4.1. Plant Material and Methodology

**Figure 4:**
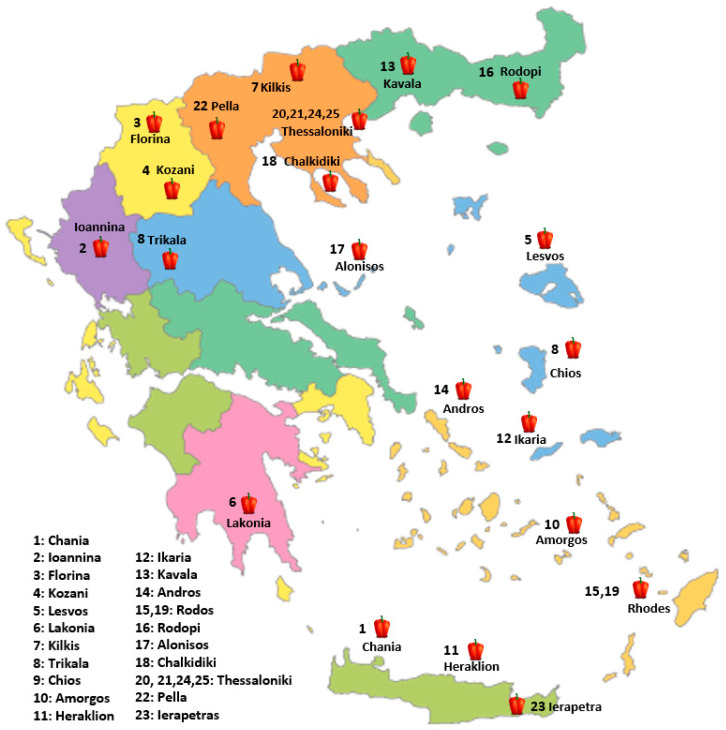
Geographic distribution of the evaluated pepper (*C. annuum* L.) genotypes across Greece. The numbers correspond to collection sites for landraces or the responsible breeding institutes for commercial varieties (20, 21, 23, 24, 25).

**Figure 5:**
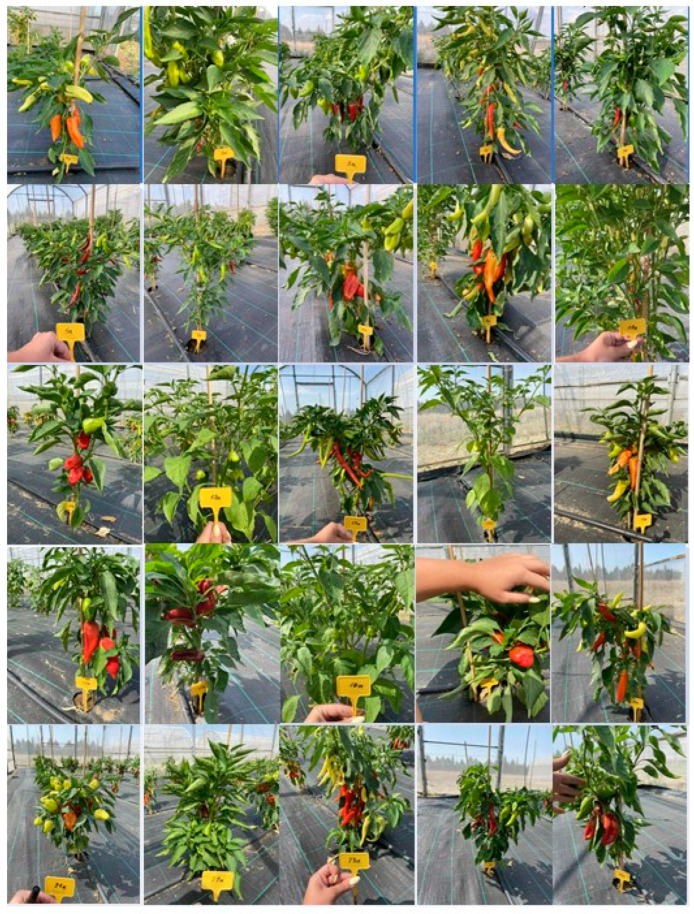
Evaluated 25 pepper (*C. annuum* L.) genotypes across Greece.

In the present study, twenty-five pepper genotypes and commercial varieties were evaluated for their performance under a low-input cultivation system. Nineteen (19) Greek traditional pepper landraces, four (4) commercial varieties derived from local populations and one (1) conservation variety. The plant material used in this study is a subset of the pepper germplasm collection of the Greek GeneBank (GGB), the Institute of Plant Breeding and Genetic Resources (IPGRB) of the Hellenic Agricultural Organization-DIMITRA (ELGO-DIMITRA), in Thermi, Thessaloniki and the Institute of Olive Tree, Subtropical Plants and Viticulture (IOSV) of the Hellenic Agricultural Organization-DIMITRA (ELGO-DIMITRA), in Chania, Crete (Table 12, Figure 4). The experiment was conducted during the spring and summer of 2024 at the facilities of the Greek Genebank (Figure 5). The twenty-five evaluated pepper genotypes were planted in a single-row system at net houses, and a randomized complete block design (RCBD) was applied with three replicates. Each replicate consisted of ten plants (a total thirty plants per genetic material) in a single steam cultivation system. The cultivation was conducted under a low-input organic farming system. Soil fertility was maintained through the application of composted sheep manure (20 t/ha), while pest and disease management relied on copper-based products and potassium salts of fatty acids, both of which are permitted under EU organic farming regulations. Weed management was achieved by installing black plastic mulch, into which holes were manually perforated to allow for the transplanting of young pepper seedlings. The planting distance was 50 cm between plants within rows and 1 m between rows. Drip irrigation was applied every two days to maintain the appropriate water supply. Observations and measurements were recorded on an individual plant basis for each variety, and yield, descriptive, and qualitative characteristics were systematically assessed.

### 4.2. Traits Evaluated

#### 4.2.1. Descriptive Characteristics

Descriptive characteristics were estimated according to the UPOV system involving plants, leaves, flowers, and fruits of all the twenty-five genotypes studied (https://www.upov.int/edocs/mdocs/upov/en/twv_54/tg_76_9_proj_2.pdf, accessed on 13 July 2025). More specifically thirty-nine characteristics were estimated: anthocyanin coloration of hypocotyl (1: absent, 2: present), shortened internodes (1: absent, 9: present), number of internodes between the first flower and shortened internodes (1: none, 2: one to three 3: more than three), plant height (cm), stem: length (cm), leaf length (cm), leaf width (cm), stem: intensity of anthocyanin coloration of nodes (1: absent or very weak, 2: weak, 3: medium, 4: strong, 5: very strong), leaf blade: shape (1: lanceolate, 2: ovate, 3: broad elliptic), leaf blade: intensity of green color (1: very light, 3: light, 5: medium, 7: dark, 9: very dark), leaf blade: undulation of margin (1: absent, 3: weak, 5: medium, 7: strong, 9: very strong), leaf blade: blistering (1: very weak, 3: weak, 5: medium, 7: strong, 9: very strong), leaf blade: glossiness (1: very weak, 3: weak, 5: medium, 7: strong, 9: very strong), ornamental varieties (1: yes, 9: no), flower: attitude of peduncle (1: predominantly erect, 2: predominantly semi-drooping, 3: predominantly drooping), flower: color (1: white, 2: light purple, 3: medium purple, 4: dark purple), only varieties with immature fruit: color green or purple: immature fruit: intensity of color (3: light, 5: medium, 7: dark, 9: very dark), immature fruit: anthocyanin coloration (1: absent or weak, 2: medium, 3: strong), fruit: attitude (1: erect, 2: horizontal, 3: drooping), fruit: length (cm), fruit: diameter (cm), fruit: length/diameter, fruit: shape in longitudinal section (1: oblate, 2: circular, 3: heart-shaped, 4: square, 5: rectangular, 6: trapezoidal, 7: triangular, 8: bullet-shaped), fruit: sinuation of pericarp at basal part (1: absent, 3: weak, 5: medium, 7: strong, 9: very strong), fruit: sinuation of pericarp excluding basal part (1: absent, 3: weak, 5: medium, 7: strong, 9: very strong), fruit: shape of apex (1: very acute, 2: moderately acute, 3: rounded, 4: moderately depressed, 5: very depressed), fruit: texture of surface (1: smooth, 2: slightly wrinkled, 3: strongly wrinkled), fruit: intensity of color (1: light, 5: medium, 3: dark), fruit: glossiness (1: very weak, 3: weak, 5: medium, 7: strong, 9: very strong), fruit: depth of peduncle cavity (1: absent, 3: shallow, 5: medium, 7: deep, 9: very deep), fruit: depth of interlocular grooves (1: absent, 3: shallow, 5: medium, 7: deep), fruit: number of locules (1: fruit: number of locules, 2: equally two and three, 3: predominantly three, 4: equally three and four, 5: predominantly four), fruit: thickness of flesh (mm), fruit: capsaicin in placenta (1: absent, 9: present), fruit: seeds (1: absent, 9: present), peduncle: length (1: very short, 3: short, 5: medium, 7: long, 9: very long), peduncle: thickness (mm), calyx: aspect (1: non enveloping, 2: enveloping), time of maturity (1: very early, 3: early, 5: medium, 7: late, 9: very late). From the thirty-nine traits evaluated, eleven are presented in the manuscript due to their agronomic significance and their discriminative power in classifying the genetic material into distinct groups.

#### 4.2.2. Yield Characteristics

Table-ripe fruit yield was estimated for each plant individually, and table-ripe fruits were counted, weighed, and graded into marketable and non-marketable categories based on their external appearance and commercial standards. Table-ripe fruit yield was recorded individually for each plant across six harvests under all cultivation systems. Earliness was assessed at 55 days after transplanting (D.A.T.), based on the cumulative yield of the first three harvests. Since marketable yield was almost equal to total yield in this work, only the total yield characteristics are presented.

#### 4.2.3. Fruit Quality and Nutritional Value Characteristics

To evaluate fruit quality and nutritional value, twelve fruits at commercial maturity (green stage) and twelve at physiological maturity (red stage) were harvested from each of the 25 genotypes and subjected to physico-chemical and nutritional analyses.

The instrumental color of the peppers was measured using a Minolta CR-300 colorimeter (Minolta, Konica Minolta GmbH, Langenhagen, Germany) in the CIEL*a*b* system. A total of five samples per genotype were analyzed immediately after harvest.

Firmness or flesh resistance was measured on the whole pepper fruits 2 mm from the top of the fruit and the maximum rupture force (N) developed during the test was determined using the puncture test performed with a Texture Analyzer (TA. HD plus-Stable Micro Systems Ltd., Surrey, UK) using a needle probe diameter 2 mm, with test speed of 2 mm s^−1^, penetration distance of 10 mm. A total of five samples per genotype were analyzed immediately after harvest.

The content of total phenolic compounds was determined by the Folin–Ciocalteau spectrophotometric method, following the methodology described by Singleton et al. [39]. The results were expressed as mg gallic acid equivalents (GAE) per 100 g of fresh weight (FW). More specifically, 2 g of fresh sample was extracted with MeOH/H2O (80/20, *v*/*v*). Standard solutions of gallic acid were prepared for the calibration curve. A stock solution of GA (5000 ppm) was used to prepare standards of 50, 100, 200, 300, and 500 mg/L, along with a blank. For each reaction mixture, 4 mL of Na2CO_3_ (7.5% *w*/*v*) and 1 mL of Folin–Ciocalteu reagent were added. Absorbance was measured at 765 nm using a UV–Visible spectrophotometer (Cary 50 Conc., Varian Inc., Palo Alto, CA, USA).

The ascorbic acid (vitamin C) of the pepper fruits was measured immediately after harvest, by macerating the sample mechanically with an establishing agent (5% metaphosphoric acid) and titrating the filtered extract against 2,6 dichlorophenolindophenol [40].

The ferric reducing antioxidant ability of plasma (FRAP) assay [41] with some modifications [42] was used to evaluate the antioxidant activity of the samples. FRAP method relies on the reduction of the TPTZ (2,4,6-tri-pyridyl-striazine)-Fe complex to the TPTZ-Fe_2_ form, with an intense blue colour and absorption maximum at 593 nm. For the FRAP assay an FRAP reagent was prepared by mixing acetate buffer (0.3 M, pH 3.6), 10 mM TPTZ in 40 mM HCl and 20 mM FeCl_3_ at 10:1:1 (*v*/*v*/*v*) ratio. FRAP assay is a nonspecific method and the absorption alterations reflect the total reducing power of all the antioxidant substances found in the test solution. FRAP standard solutions were prepared using ferrous sulfate heptahydrate (FeSO_4_·7H_2_O, Sigma-Aldrich). A fresh 1 mM stock solution of FeSO_4_·7H_2_O (278.01 mg L^−1^) was prepared in deionized water immediately before use and protected from air/light to minimize oxidation. A series of working standards were made by serial dilution of the stock to give 0, 50, 100, 200, 400, 600 and 800 µM Fe(II). For each standard, 100 µL standard was mixed with 3 mL FRAP reagent, incubated at 37 °C for 30 min, and absorbance read at 593 nm. A calibration curve of absorbance (A_593_) versus Fe(II) concentration (µM) was constructed by linear regression. Concentration in µM was converted to mass of FeSO_4_·7H_2_O (µg mL^−1^) using the molecular weight of FeSO_4_·7H_2_O (278.01 g mol^−1^) with the relation: FRAP (µg/g^−1^ FW) = conc (µM) × MW × 10^−3^/g fw, A triplicate of each treatment was analysed spectrophotometrically.

Total flavonoid content was quantified following the method of [43], with minor modifications. More specifically, we reduced the sample size to 3 g of fresh tissue, used 80% (*v*/*v*) methanol instead of 95% ethanol for extraction, and optimized reagent volumes to fit a microplate spectrophotometer format. Specifically, 0.5 mL of extract was mixed with 2 mL of distilled water and 0.15 mL of 5% NaNO_2_; after 5 min, 0.15 mL of 10% AlCl_3_ was added, followed by 1 mL of 1 M NaOH after 6 min, and the final volume was adjusted to 5 mL with distilled water. Absorbance was measured at 510 nm. These modifications were introduced to improve pigment solubility in our samples and to allow efficient use of smaller extract volumes. Calibration was performed with rutin standards (0–100 µg mL^−1^ in 80% methanol). Results were expressed as mg rutin equivalents per g fresh weight (mg RE g^−1^ FW). Fresh tissue (3 g) was extracted in 80% (*v*/*v*) methanol, and the flavonoid concentration was determined colorimetrically. Rutin was used as the calibration standard, and results were expressed as milligrams of rutin equivalents per gram of fresh weight (mg RE g^−1^ FW). In our assay, rutin was used as the reference compound to construct the calibration curve. A stock solution of rutin (1 mg mL^−1^) was prepared in 80% (*v*/*v*) methanol. From this, working standard solutions were prepared in the range of 0–100 µg mL^−1^. The calibration curve was generated by plotting absorbance against rutin concentration, and the flavonoid content of samples was expressed as mg rutin equivalents per g fresh weight (mg RE g^−1^ FW). All measurements were performed in eight biological replicates, and the mean values are reported.

The concentrations of porphyrin intermediates were quantified spectrophotometrically based on their characteristic absorption peaks using a UV-Vis spectrophotometer (UV-1800, Shimadzu Corp., Kyoto, Japan). Total porphyrins were determined by measuring optical density (OD) at 575, 590, and 628 nm, which are the absorption peaks of protoporphyrin, magnesium-protoporphyrin, and proto-chlorophyllide, respectively. The sum of the above determines the total porphyrin concentration as follows: (A) Protoporphyrin = [(12.25 × A665 − 2.55 × A649) × volume of supernatant (mL) ×dilution factor/sample weight (g)]/892 × 1000, (B) Mg-Protoporphyrin = [(20.31 × A649 − 4.91 × A665) × volume of supernatant (mL) ×dilution factor/sample weight (g)]/906 × 1000, (C) Protochlorophyllide = [(196.25 × A575 − 46.6 × A590 − 58.68 × A628) + (61.81 ×A590 − 23.77 × A575 − 3.55 × A628) + (42.59 × A628 − 34.32 × A575 − 7.25 × A590)] × dilution factor/sample weight (g)] Total porphyrins = (A) + (B) + (C). This formula is based on specific extinction coefficients of pigments at defined wavelengths, used to resolve overlapping absorbance spectra of different porphyrin intermediates. Moreover, absorbance values measured at 665 nm and 649 nm, respectively. These wavelengths are chosen because protoporphyrin and related intermediates have maximum absorption there. 12.25 and 2.55 are specific absorption coefficients (sometimes called “correction factors”) derived from calibration curves of pure standards. They allow the separation of the overlapping absorbance contributions of protoporphyrin at 665 and 649 nm. V, is the final extract volume (mL) after pigment extraction, DF is the dilution factor, applied if the extract was diluted before measurement, 892 is the molecular weight (g mol^−1^) of protoporphyrin IX, used to convert absorbance data into molar concentration and W indicates the fresh weight (g FW) of the plant tissue used for extraction. The final unit (nmol g^−1^ FW) represents the normalized pigment concentration, expressed per gram of fresh tissue.

#### 4.2.4. Statistical Analysis

All the morphological, agronomic, and nutritional traits measured were subjected to analysis of variance (ANOVA), and mean comparisons were performed using Duncan’s Multiple Range Test at a significance level of *p* ≤ 0.05, to identify statistically significant differences among the pepper genotypes. To explore the phenotypic diversity and potential phylogenetic relationships among 25 pepper (*Capsicum annuum* L.) genetic materials, a multivariate statistical approach was employed. All recorded variables, including morphological descriptors, agronomic performance traits, and fruit nutritional attributes—were subjected to Principal Component Analysis (PCA) in order to reduce dimensionality and highlight the main axes of variation among genotypes. Subsequently, a hierarchical cluster analysis was performed using the Average Linkage (Between Groups) method and Euclidean distance as the similarity measure. The resulting dendrogram provided a graphical representation of the clustering patterns among the genotypes. All analyses were performed using IBM SPSS Statistics (V28) [44]. Heatmap was generated for using (version 1.0.12) (Kolde, R 2019) package in R [45].

## 5. Conclusions

The comprehensive evaluation of 25 Greek local and commercial pepper varieties revealed remarkable genetic diversity across morphological, agronomic, and nutritional traits, confirming the exceptional value of Greek landraces as genetic resources for sustainable agriculture. The wide variation observed in plant architecture, fruit morphology, yield components, and bioactive compounds such as phenolics, and vitamin C demonstrates their potential for breeding programs aiming to enhance productivity, nutritional quality, and resilience under low-input conditions. Genotypes like ‘Skopos’ and ‘Mesoropi’ combined high yield with strong antioxidant capacity, while others such as ‘Pogoniou’, ‘Lyra’, and ‘Kantanou’ displayed outstanding nutritional profiles suitable for functional food or nutraceutical exploitation.

The distinctive geomorphology of Greece—with its diverse microclimates ranging from the cold mountainous north to the warm southern regions, and from high-altitude areas to isolated islands—appears to have played a decisive role in shaping this variability, enabling the development of distinct and locally adapted pepper populations. Greece thus represents a unique geographic mosaic rich in genetic diversity, where centuries of adaptation and isolation have fostered the evolution of valuable landraces ideally suited for modern breeding and sustainable cultivation.

These findings highlight that several non-improved landraces can serve as baseline material for developing cultivars resilient to climate change and capable of meeting consumer demands for high-quality produce. Integrating this germplasm into molecular characterization, genome-assisted, and participatory breeding schemes will accelerate the utilization of local biodiversity. Overall, the conservation and sustainable exploitation of Greek pepper landraces should be viewed not only as a cultural heritage priority but also as a strategic pathway toward climate-smart, resilient, and nutritionally enriched agri-food systems.

## Figures and Tables

**Table 1:** Plant Morphological Characteristics.

A/A	Entry	Plant Height (cm)	Stem: Length (cm)	Leaf Length (cm)	Leaf Width (cm)
1	Kantanou	30.00 _bcde_	24.00 _bc_	6.99 _ab_	3.54 _bc_
2	Pogoniou	29.00 _bcde_	24.00 _bc_	6.95 _ab_	3.52 _bc_
3	Skopos	27.33 _cdef_	24.00 _bc_	7.71 _a_	3.58 _b_
4	Anarahi	20.00 _gh_	15.00 _e_	7.43 _a_	3.41 _bcd_
5	Evriaki	32.00 _abc_	23.00 _bcd_	6.40 _abc_	3.18 _bcd_
6	Lyra	37.33 _a_	29.00 _a_	6.13 _abc_	3.40 _bcd_
7	Filyria	31.33 _abcd_	23.00 _bcd_	5.43 _bc_	2.52 _d_
8	Lygaria	31.00 _abcd_	27.00 _ab_	7.23 _ab_	3.90 _ab_
9	Sykousis	25.00 _defg_	21.00 _de_	7.23 _ab_	3.85 _ab_
10	Lagada	14.67 _h_	8.00 _e_	4.73 _c_	2.61 _cd_
11	Lyttos	27.00 _cdef_	22.00 _cd_	7.35 _a_	4.68 _a_
12	Akamatra	16.33 _h_	11.00 _e_	6.15 _abc_	3.85 _ab_
13	Mesoropi	33.00 _abc_	27.00 _ab_	6.33 _abc_	3.30 _bcd_
14	Ano Gavrio	28.33 _bcde_	22.00 _cd_	6.60 _ab_	3.83 _ab_
15	Arnaouti	32.67 _abc_	24.00 _bc_	6.27 _abc_	3.35 _bcd_
16	Kentavros	24.00 _efg_	20.00 _de_	6.15 _abc_	3.28 _bcd_
17	Votsi	30.33 _bcde_	23.00 _bcd_	6.86 _ab_	3.63 _b_
18	Galatista	21.00 _fgh_	14.00 _e_	7.62 _a_	4.67 _a_
19	Apollonas	30.33 _bcde_	25.00 _bc_	6.78 _ab_	3.83 _ab_
20	P13	34.67 _ab_	29.00 _a_	6.75 _ab_	3.28 _bcd_
21	P14	29.33 _bcde_	24.00 _bc_	7.56 _a_	3.64 _b_
22	Bachovitiki	28.00 _cde_	27.00 _ab_	6.64 _ab_	3.39 _bcd_
23	Ierapetras	28.00 _cde_	20.00 _de_	6.07 _abc_	3.11 _bcd_
24	Platika Florinis	37.00 _a_	31.00 _a_	6.47 _abc_	3.76 _ab_
25	Florinis	32.00 _abc_	27.00 _ab_	6.62 _ab_	3.76 _ab_

Different letters within the same column indicate statistically significant differences among genotypes according to Duncan’s Multiple Range Test (*p* ≤ 0.05).

**Table 2:** Fruit Dimensions and Shape.

A/A	Entry	Fruit: Length (cm)	Fruit: Diameter (cm)	Fruit: Length/ Diameter	Fruit: Shape in Longitudinal Section *
1	Kantanou	10.50 _cd_	2.59 _ef_	4.80 _fg_	6.00 _bc_
2	Pogoniou	11.35 _cd_	1.78 _gh_	6.39 _e_	7.00 _ab_
3	Skopos	9.775 _de_	3.21 _cd_	3.10 _hi_	7.00 _ab_
4	Anarahi	13.94 _b_	1.65 _ghi_	8.71 _bc_	7.00 _ab_
5	Evriaki	9.291 _de_	1.90 _gh_	4.75 _fg_	7.00 _ab_
6	Lyra	9.258 _de_	1.24 _ijk_	7.55 _d_	7.00 _ab_
7	Filyria	7.983 _ef_	1.07 _jk_	7.85 _cd_	7.00 _ab_
8	Lygaria	11.05 _cd_	3.41 _c_	2.35 _ijk_	5.00 _c_
9	Sykousis	13.99 _b_	2.58 _ef_	5.60 _ef_	7.00 _ab_
10	Lagada	3.20 _i_	0.80 _k_	3.96 _gh_	9.00 _a_
11	Lyttos	6.81 _fg_	4.07 _b_	1.63 _jk_	5.00 _c_
12	Akamatra	3.53 _i_	2.18 _fg_	1.60 _jk_	5.00 _c_
13	Mesoropi	16.11 _a_	1.52 _hij_	10.50 _a_	7.00 _ab_
14	Ano Gavrio	3.82 _hi_	2.47 _ef_	1.57 _jk_	7.00 _ab_
15	Arnaouti	10.21 _cd_	2.68 _def_	3.80 _gh_	7.00 _ab_
16	Kentavros	11.12 _cd_	2.72 _def_	4.28 _gh_	7.00 _ab_
17	Votsi	10.86 _cd_	2.66 _ef_	4.16 _gh_	6.00 _bc_
18	Galatista	3.52 _i_	2.76 _de_	1.27 _k_	5.00 _c_
19	Apollonas	5.56 _gh_	5.04 _a_	1.39 _jk_	4.00 _c_
20	P13	12.10 _c_	1.90 _gh_	6.45 _e_	7.00 _ab_
21	P14	7.34 _fg_	5.02 _a_	1.43 _jk_	5.00 _c_
22	Bachovitiki	5.88 _g_	2.65 _ef_	2.16 _ijk_	6.00 _bc_
23	Ierapetras	14.52 _ab_	1.51 _hij_	9.67 _ab_	7.00 _ab_
24	Platika Florinis	10.57 _cd_	2.80 _de_	3.80 _gh_	7.00 _ab_
25	Florinis	11.31 _cd_	4.41 _b_	2.53 _ij_	6.00 _bc_

* 4: square, 5: rectangular, 6: trapezoidal, 7: triangular, 9: horn-shaped. Different letters within the same column indicate statistically significant differences among genotypes according to Duncan’s Multiple Range Test (*p* ≤ 0.05).

**Table 3:** Internal Fruit Characteristics.

A/A	Entry	Fruit: Number of Locules *	Fruit: Thickness of Flesh (mm)	Fruit: Capsaicin in the Placenta (1: Absent. 9: Present)
1	Kantanou	1.00 _c_	5.00 _bc_	1.00 _c_
2	Pogoniou	3.00 _b_	3.00 _cd_	9.00 _a_
3	Skopos	1.00 _c_	7.00 _ab_	1.00 _c_
4	Anarahi	1.00 _c_	5.00 _bc_	9.00 _a_
5	Evriaki	1.00 _c_	1.00 _d_	9.00 _a_
6	Lyra	3.00 _b_	1.00 _d_	9.00 _a_
7	Filyria	1.00 _c_	1.00 _d_	9.00 _a_
8	Lygaria	5.00 _a_	9.00 _a_	1.00 _c_
9	Sykousis	1.00 _c_	7.00 _ab_	9.00 _a_
10	Lagada	2.00 _c_	3.00 _cd_	9.00 _a_
11	Lyttos	5.00 _a_	3.00 _cd_	1.00 _c_
12	Akamatra	5.00 _a_	5.00 _bc_	1.00 _c_
13	Mesoropi	3.00 _b_	1.00 _d_	1.00 _c_
14	Ano Gavrio	1.00 _c_	3.00 _cd_	1.00 _c_
15	Arnaouti	3.00 _b_	3.00 _cd_	1.00 _c_
16	Kentavros	1.00 _c_	7.00 _ab_	9.00 _a_
17	Votsi	2.00 _c_	1.00 _d_	1.00 _c_
18	Galatista	5.00 _a_	5.00 _bc_	1.00 _c_
19	Apollonas	5.00 _a_	9.00 _a_	1.00 _c_
20	P13	2.00 _c_	5.00 _bc_	1.00 _c_
21	P14	5.00 _a_	5.00 _bc_	1.00 _c_
22	Bachovitiki	1.00 _c_	3.00 _cd_	1.00 _c_
23	Ierapetras	2.00 _c_	5.00 _bc_	3.00 _b_
24	Platika Florinis	3.00 _b_	7.00 _ab_	1.00 _c_
25	Florinis	3.00 _b_	7.00 _ab_	1.00 _c_

* 1: Fruit: number of locules, 2: equally two and three, 3: predominantly three, and 5: predominantly four. Different letters within the same column indicate statistically significant differences among genotypes according to Duncan’s Multiple Range Test (*p* ≤ 0.05).

**Table 4:** Early Yield Traits.

A/A	Entry	Early Fruit Number	Early Plant Yield (g)	Early Fruit Weight (g)
1	Kantanou	4.51 _bcde_	75.10 _abcd_	18.04 _bcdef_
2	Pogoniou	3.75 _bcde_	58.27 _bcd_	15.38 _cdef_
3	Skopos	4.95 _bcde_	106.10 _abcd_	30.01 _abcde_
4	Anarahi	3.20 _cde_	76.40 _abcd_	24.25 _abcdef_
5	Evriaki	3.77 _bcde_	72.56 _abcd_	16.48 _bcdef_
6	Lyra	6.28 _abcd_	43.25 _bcd_	8.41 _def_
7	Filyria	9.33 _ab_	45.37 _bcd_	9.60 _def_
8	Lygaria	2.57 _de_	120.10 _ab_	46.51 _a_
9	Sykousis	3.50 _bcde_	108.70 _abc_	28.35 _abcde_
10	Lagada	4.91 _bcde_	12.07 _cd_	5.32 _ef_
11	Lyttos	2.11 _cde_	61.95 _abcd_	24.46 _abcdef_
12	Akamatra	9.03 _abc_	15.57 _d_	1.66 _f_
13	Mesoropi	10.80 _a_	163.40 _a_	16.48 _bcdef_
14	Ano Gavrio	1.67 _de_	10.99 _cd_	7.23 _def_
15	Arnaouti	3.00 _cde_	89.05 _abcd_	27.96 _abcde_
16	Kentavros	4.77 _bcde_	142.30 _ab_	34.13 _abcd_
17	Votsi	5.65 _abcde_	122.10 _ab_	22.84 _abcdef_
18	Galatista	0.00 _e_	0.00 _d_	1.11 _f_
19	Apollonas	2.51 _de_	99.92 _abcd_	40.16 _abc_
20	P13	2.66 _cde_	65.64 _abcd_	26.89 _abcdef_
21	P14	2.63 _cde_	115.90 _ab_	42.63 _ab_
22	Bachovitiki	2.25 _cde_	36.46 _bcd_	16.30 _bcdef_
23	Ierapetras	5.13 _bcde_	67.10 _abcd_	13.62 _cdef_
24	Platika Florinis	3.00 _cde_	71.83 _abcd_	26.68 _abcdef_
25	Florinis	2.27 _de_	82.93 _abcd_	41.62 _ab_

Different letters within the same column indicate statistically significant differences among genotypes according to Duncan’s Multiple Range Test (*p* ≤ 0.05).

**Table 5:** Total Yield Traits.

A/A	Entry	Total Fruit Number	Total Plant Yield (g)	Total Fruit Weight (g)
1	Kantanou	13.16 _de_	216.81 _bc_	16.46 _bcdefghi_
2	Pogoniou	10.01 _cde_	140.06 _bc_	14.58 _efghi_
3	Skopos	15.50 _bcde_	627.74 _a_	39.01 _ab_
4	Anarahi	11.49 _cde_	243.95 _bc_	21.45 _bcdefgh_
5	Evriaki	15.72 _bcde_	276.59 _bc_	16.80 _cdefghi_
6	Lyra	28.41 _abc_	183.44 _bc_	9.10 _ghi_
7	Filyria	30.94 _ab_	142.96 _bc_	4.99 _hi_
8	Lygaria	11.22 _cde_	401.93 _ab_	34.87 _abcd_
9	Sykousis	10.66 _cde_	263.90 _bc_	26.95 _abcdefg_
10	Lagada	25.22 _abcd_	41.11 _c_	5.31 _hi_
11	Lyttos	8.249 _de_	193.05 _bc_	26.17 _abcdefg_
12	Akamatra	14.92 _bcde_	15.38 _c_	2.42 _i_
13	Mesoropi	36.12 _a_	455.57 _ab_	13.96 _efghi_
14	Ano Gavrio	7.96 _e_	98.19 _c_	6.53 _ghi_
15	Arnaouti	8.94 _de_	178.09 _bc_	19.36 _bcdefghi_
16	Kentavros	14.89 _bcde_	339.49 _bc_	26.82 _abcdefg_
17	Votsi	16.88 _bcde_	328.00 _bc_	21.42 _bcdefgh_
18	Galatista	0.25 _e_	28.86 _c_	11.19 _fghi_
19	Apollonas	9.56 _cde_	290.48 _bc_	33.59 _abcde_
20	P13	10.13 _cde_	206.46 _bc_	15.87 _defghi_
21	P14	8.94 _de_	339.39 _bc_	41.62 _a_
22	Bachovitiki	8.17 _de_	126.26 _bc_	19.88 _bcdefghi_
23	Ierapetras	20.85 _abcde_	251.85 _bc_	12.70 _fghi_
24	Platika Florinis	10.27 _cde_	220.92 _bc_	29.02 _abcdef_
25	Florinis	9.10 _de_	304.05 _bc_	36.36 _abc_

Different letters within the same column indicate statistically significant differences among genotypes according to Duncan’s Multiple Range Test (*p* ≤ 0.05).

**Table 6:** Green Fruit Color Parameters (measured at commercial maturity).

A/A	Entry	Green Fruit: L*	Green Fruit: a*	Green Fruit: b*
1	Kantanou	47.87 _hij_	21.38 _abcd_	33.25 _efgh_
2	Pogoniou	51.01 _fghi_	22.47 _ab_	41.58 _bcdef_
3	Skopos	52.27 _efgh_	22.45 _ab_	35.10 _defgh_
4	Anarahi	68.34 _a_	13.04 _efgh_	31.96 _fgh_
5	Evriaki	43.06 _jk_	6.08 _h_	19.83 _i_
6	Lyra	53.76 _efgh_	21.16 _abcd_	44.81 _bcd_
7	Filyria	49.13 _ghij_	23.82 _a_	38.45 _cdefg_
8	Lygaria	55.31 _defg_	21.59 _abcd_	41.57 _bcdef_
9	Sykousis	57.59 _def_	12.86 _efgh_	30.83 _gh_
10	Lagada	66.20 _ab_	7.50 _gh_	38.39 _cdefg_
11	Lyttos	44.13 _jk_	20.35 _abcd_	28.29 _ghi_
12	Akamatra	61.85 _abcd_	19.14 _abcdef_	49.01 _ab_
13	Mesoropi	55.97 _def_	21.67 _abc_	45.30 _bc_
14	Ano Gavrio	65.10 _abc_	16.41 _bcdef_	56.82 _a_
15	Arnaouti	60.66 _bcd_	22.49 _ab_	46.26 _bc_
16	Kentavros	55.65 _defg_	14.35 _defg_	42.33 _bcde_
17	Votsi	59.51 _cde_	21.60 _abcd_	45.36 _bc_
18	Galatista	59.43 _cde_	19.59 _abcde_	49.94 _ab_
19	Apollonas	45.15 _ijk_	18.99 _abcdef_	30.76 _gh_
20	P13	67.59 _a_	15.10 _cdef_	37.50 _cdefg_
21	P14	52.68 _efgh_	20.37 _abcd_	37.57 _cdefg_
22	Bachovitiki	38.96 _k_	17.58 _abcdef_	20.82 _i_
23	Ierapetras	67.13 _ab_	11.94 _fgh_	29.30 _ghi_
24	Platika Florinis	51.02 _fghi_	19.89 _abcde_	37.65 _cdefg_
25	Florinis	43.56 _jk_	20.75 _abcd_	27.04 _hi_

Different letters within the same column indicate statistically significant differences among genotypes according to Duncan’s Multiple Range Test (*p* ≤ 0.05).

**Table 7:** Green Fruit Firmness, Phenolics and Vitamin C (measured at commercial maturity).

A/A	Entry	Green Fruit: Texture (Newton)	Green Fruit: Gallic Equivalent (mg/100 g FW Tissue)	Green Fruit: Vit C (mg/100 g FW) *n* = 3
1	Kantanou	7.88 _cde_	61.20 _def_	51.01 _efg_
2	Pogoniou	10.40 _bcd_	134.00 _a_	128.10 _bc_
3	Skopos	8.34 _cde_	88.77 _cde_	57.95 _efg_
4	Anarahi	5.06 _f_	47.46 _fg_	39.47 _fgh_
5	Evriaki	10.70 _bcd_	74.99 _def_	86.00 _def_
6	Lyra	6.75 _def_	129.80 _ab_	52.55 _efg_
7	Filyria	10.00 _bcd_	120.10 _bcd_	143.00 _b_
8	Lygaria	6.02 _def_	50.82 _efg_	205.00 _a_
9	Sykousis	8.65 _cde_	38.51 _gh_	51.26 _efg_
10	Lagada	8.09 _cde_	137.80 _a_	25.83 _gh_
11	Lyttos	9.13 _bcd_	67.96 _def_	116.40 _cd_
12	Akamatra	10.60 _bcd_	53.27 _efg_	18.85 _i_
13	Mesoropi	5.86 _ef_	57.42 _defg_	19.51 _hi_
14	Ano Gavrio	13.50 _a_	65.48 _def_	33.87 _gh_
15	Arnaouti	5.77 _ef_	28.95 _gh_	38.64 _fgh_
16	Kentavros	12.20 _ab_	47.02 _fg_	18.85 _i_
17	Votsi	8.32 _cde_	90.55 _cde_	26.01 _gh_
18	Galatista	11.60 _ab_	34.79 _gh_	24.90 _ghi_
19	Apollonas	14.90 _a_	65.46 _def_	60.80 _efg_
20	P13	8.71 _cde_	37.52 _gh_	19.04 _hi_
21	P14	7.36 _def_	46.49 _fg_	42.56 _fgh_
22	Bachovitiki	7.58 _def_	42.17 _fg_	25.14 _gh_
23	Ierapetras	5.63 _ef_	61.44 _def_	32.52 _gh_
24	Platika Florinis	8.85 _cde_	43.25 _fg_	45.52 _fgh_
25	Florinis	6.81 _def_	24.34 _h_	38.07 _fgh_

Different letters within the same column indicate statistically significant differences among genotypes according to Duncan’s Multiple Range Test (*p* ≤ 0.05).

**Table 8:** FRAP, Flavonoids, Porphyrins (measured at commercial maturity).

A/A	Entry	Green Fruit: FRAP (μg/g FW)	Green Fruit: Flavonoids (mg/100 g FW)	Green Fruit: Porphyrins (mg/g FW)
1	Kantanou	470.29 _a_	13.86 _a_	0.53 _a_
2	Pogoniou	794.39 _a_	24.95 _a_	0.83 _a_
3	Skopos	648.25 _a_	18.80 _a_	0.67 _a_
4	Anarahi	703.92 _a_	19.48 _a_	0.64 _a_
5	Evriaki	655.84 _a_	16.40 _a_	0.56 _a_
6	Lyra	668.96 _a_	26.41 _a_	0.79 _a_
7	Filyria	1079.50 _a_	26.30 _a_	0.82 _a_
8	Lygaria	424.80 _a_	13.06 _a_	0.41 _a_
9	Sykousis	294.12 _a_	10.55 _a_	0.39 _a_
10	Lagada	968.23 _a_	27.02 _a_	0.77 _a_
11	Lyttos	668.82 _a_	16.05 _a_	0.64 _a_
12	Akamatra	152.30 _a_	12.87 _a_	0.46 _a_
13	Mesoropi	695.56 _a_	11.27 _a_	0.41 _a_
14	Ano Gavrio	399.23 _a_	13.53 _a_	0.48 _a_
15	Arnaouti	577.01 _a_	10.96 _a_	0.42 _a_
16	Kentavros	475.45 _a_	15.54 _a_	0.54 _a_
17	Votsi	737.23 _a_	19.34 _a_	0.65 _a_
18	Galatista	330.98 _a_	9.29 _a_	0.34 _a_
19	Apollonas	793.02 _a_	19.55 _a_	0.23 _a_
20	P13	466.36 _a_	12.25 _a_	0.47 _a_
21	P14	377.22 _a_	11.87 _a_	0.40 _a_
22	Bachovitiki	547.43 _a_	18.05 _a_	0.58 _a_
23	Ierapetras	496.09 _a_	13.35 _a_	0.51 _a_
24	Platika Florinis	642.17 _a_	18.95 _a_	0.57 _a_
25	Florinis	573.25 _a_	15.50 _a_	0.51 _a_

Different letters within the same column indicate statistically significant differences among genotypes according to Duncan’s Multiple Range Test (*p* ≤ 0.05).

**Table 9:** Red Fruit Color Parameters (measured at physiological maturity).

A/A	Entry	Red Fruit: L*	Red Fruit: a*	Red Fruit: b*
1	Kantanou	38.71 _def_	37.33 _abcde_	26.33 _bcdefg_
2	Pogoniou	35.42 _fg_	36.27 _abcde_	18.70 _fg_
3	Skopos	36.83 _efg_	34.33 _abcde_	17.15 _g_
4	Anarahi	41.30 _bcd_	35.49 _abcde_	32.26 _bcd_
5	Evriaki	38.46 _def_	33.09 _bcde_	18.03 _fg_
6	Lyra	34.34 _g_	35.11 _abcde_	21.01 _defg_
7	Filyria	38.81 _cdef_	38.88 _abc_	28.96 _bcdefg_
8	Lygaria	35.21 _fg_	34.14 _abcde_	26.86 _bcdefg_
9	Sykousis	36.22 _efg_	31.75 _cde_	19.16 _fg_
10	Lagada	42.46 _bc_	41.40 _a_	31.31 _bcde_
11	Lyttos	39.58 _cde_	38.31 _abcd_	26.46 _bcdefg_
12	Akamatra	46.84 _a_	35.78 _abcde_	46.17 _a_
13	Mesoropi	39.32 _cde_	41.14 _a_	25.44 _bcdefg_
14	Ano Gavrio	47.30 _a_	37.77 _abcde_	37.28 _ab_
15	Arnaouti	42.41 _bc_	40.77 _a_	29.89 _bcdef_
16	Kentavros	37.91 _defg_	30.28 _e_	22.37 _cdefg_
17	Votsi	38.39 _def_	38.35 _abc_	19.41 _efg_
18	Galatista	43.43 _b_	35.51 _abcde_	33.34 _bc_
19	Apollonas	40.93 _bcd_	36.45 _abcde_	26.53 _bcdefg_
20	P13	43.23 _b_	40.33 _ab_	33.32 _bc_
21	P14	39.84 _bcde_	35.17 _abcde_	23.65 _cdefg_
22	Bachovitiki	37.92 _defg_	40.33 _ab_	22.76 _cdefg_
23	Ierapetras	38.08 _def_	40.74 _a_	23.53 _cdefg_
24	Platika Florinis	38.61 _def_	30.79 _de_	22.50 _cdefg_
25	Florinis	36.97 _efg_	36.65 _abcde_	22.66 _cdefg_

Different letters within the same column indicate statistically significant differences among genotypes according to Duncan’s Multiple Range Test (*p* ≤ 0.05).

**Table 10:** Red Fruit Texture, Phenolics And Vitamin C (measured at physiological maturity).

A/A	Entry	Red Fruit: Texture (Newton)	Red Fruit: Gallic Equivalent (mg/100 g FW Tissue)	Red Fruit: Vit C (mg/100 g FW)
1	Kantanou	5.86 _def_	104.62 _bcdef_	120.89 _cde_
2	Pogoniou	7.55 _bcd_	155.61 _ab_	167.59 _a_
3	Skopos	4.94 _ef_	165.47 _a_	160.04 _ab_
4	Anarahi	5.58 _def_	120.48 _abcdef_	102.75 _def_
5	Evriaki	8.05 _bcd_	131.37 _abcde_	145.78 _bcd_
6	Lyra	10.00 _ab_	127.35 _abcde_	105.67 _def_
7	Filyria	3.31 _ef_	149.12 _abc_	150.24 _bcd_
8	Lygaria	4.71 _ef_	100.48 _bcdef_	102.75 _def_
9	Sykousis	7.56 _bcd_	136.29 _abcd_	148.19 _bcd_
10	Lagada	15.50 _a_	117.92 _abcdef_	78.58 _efg_
11	Lyttos	8.97 _bcd_	145.27 _abc_	136.99 _cde_
12	Akamatra	6.98 _cde_	73.09 _ef_	59.96 _efg_
13	Mesoropi	5.85 _def_	61.42 _f_	68.17 _efg_
14	Ano Gavrio	6.31 _cde_	71.493 _ef_	30.37 _fg_
15	Arnaouti	3.38 _ef_	78.20 _def_	68.86 _efg_
16	Kentavros	6.60 _cde_	89.01 _cdef_	89.78 _efg_
17	Votsi	8.63 _bcd_	135.61 _abcd_	118.94 _cde_
18	Galatista	7.32 _cde_	93.92 _cdef_	59.28 _efg_
19	Apollonas	10.10 _ab_	93.57 _cdef_	41.10 _fg_
20	P13	5.86 _def_	78.32 _def_	44.73 _fg_
21	P14	6.75 _cde_	140.72 _abc_	119.71 _cde_
22	Bachovitiki	5.79 _def_	74.00 _ef_	24.42 _g_
23	Ierapetras	6.29 _cde_	100.61 _bcdef_	103.26 _def_
24	Platika Florinis	1.79 _f_	104.01 _bcdef_	113.84 _cdef_
25	Florinis	6.71 _cde_	100.63 _bcdef_	54.40 _fg_

Different letters within the same column indicate statistically significant differences among genotypes according to Duncan’s Multiple Range Test (*p* ≤ 0.05).

**Table 11:** FRAP, Flavonoids, Porphyrins (measured at physiological maturity).

A/A	Entry	Red Fruit: FRAP (μg/g FW)	Red Fruit: Flavonoids (mg/100 g FW)	Red Fruit: Porphyrins (mg/g FW)
1	Kantanou	1132.48 _a_	28.63 _a_	0.90 _a_
2	Pogoniou	890.16 _abcd_	22.73 _abcde_	0.77 _abcd_
3	Skopos	797.08 _abcde_	19.36 _cde_	0.62 _bcde_
4	Anarahi	1132.48 _a_	25.72 _abc_	0.89 _ab_
5	Evriaki	931.49 _abc_	23.10 _abcde_	0.74 _abcde_
6	Lyra	1077.65 _ab_	27.17 _ab_	0.85 _abcd_
7	Filyria	772.16 _abcde_	18.36 _cde_	0.65 _abcde_
8	Lygaria	983.55 _abc_	25.09 _abcde_	0.86 _abc_
9	Sykousis	722.86 _abcdef_	17.78 _e_	0.62 _bcde_
10	Lagada	972.26 _abc_	25.53 _abcd_	0.83 _abcd_
11	Lyttos	1152.13 _a_	23.15 _abcde_	0.82 _abcd_
12	Akamatra	676.09 _abcdef_	18.11 _cde_	0.69 _abcde_
13	Mesoropi	325.99 _ef_	18.67 _cde_	0.70 _abcde_
14	Ano Gavrio	251.29 _f_	17.68 _e_	0.60 _cde_
15	Arnaouti	406.97 _def_	21.56 _abcde_	0.70 _abcde_
16	Kentavros	677.26 _abcdef_	17.33 _e_	0.48 _e_
17	Votsi	1017.19 _abc_	27.05 _ab_	0.84 _abcd_
18	Galatista	732.29 _abcdef_	18.14 _cde_	0.65 _abcde_
19	Apollonas	814.30 _abcde_	21.59 _abcde_	0.70 _abcde_
20	P13	1061.97 _abc_	25.83 _abc_	0.90 _a_
21	P14	868.95 _abcd_	20.82 _bcde_	0.73 _abcde_
22	Bachovitiki	719.21 _abcdef_	21.63 _abcde_	0.73 _abcde_
23	Ierapetras	1030.15 _abc_	22.45 _abcde_	0.73 _abcde_
24	Platika Florinis	570.51 _bcdef_	17.59 _e_	0.61 _cde_
25	Florinis	560.33 _cdef_	17.85 _de_	0.58 _de_

Different letters within the same column indicate statistically significant differences among genotypes according to Duncan’s Multiple Range Test (*p* ≤ 0.05).

**Table 12:** The information regarding the origin of the 25 studied Greek traditional and commercial pepper genotypes. GGB: Greek GeneBank, IPGRB: Institute of Plant Breeding and Genetic Resources, IOSV: Institute of Olive Tree, Subtropical Plants and Viticulture.

A/A	Entry	Collection Number/Commercial Name	Type/Category	Collection Institute/Source	Collection Site
1	Kantanou	GRC194/04	Landrace	GGB	Chania-Kantanos
2	Pogoniou	GRC1427/04	Landrace	GGB	Ioannina-Pogoni
3	Skopos	F-013/06	Landrace	GGB	Florina-Skopos
4	Anarahi	K-066/06	Landrace	GGB	Kozani-Anarahi
5	Evriaki	M-037/06	Landrace	GGB	Lesvos-Paleokipos
6	Lyra	P-173/06	Landrace	GGB	Lakonia-Lyra
7	Filyria	SK-018/06	Landrace	GGB	Kilkis-Filyria
8	Lygaria	T-323/06	Landrace	GGB	Trikala-Lygaria
9	Sykousis	X-036/06	Landrace	GGB	Chios-Ag. Georgios Sykousis
10	Lagada	ANP-055/07	Landrace	GGB	Amorgos-Lagada
11	Lyttos	HL-049/07	Landrace	GGB	Irakleion-Lyttos
12	Akamatra	IS-095/07	Landrace	GGB	Ikaria-Akamatra
13	Mesoropi	KD-079/07	Landrace	GGB	Kavala Mesoropi
14	Ano Gavrio	ATS-017/06	Landrace	GGB	Andros-Ano Gavrio
15	Arnaouti	RK-053/07	Landrace	GGB	Rodos-Ag. Isidoros
16	Kentavros	ROX-099/07	Landrace	GGB	Rodopi-Kentavros
17	Votsi	SAS-046/07	Landrace	GGB	Alonissos-Votsi
18	Galatista	XKA-115/07	Landrace	GGB	Chalkidiki-Galatista
19	Apollonas	GRC139/05	Landrace	GGB	Rodos
20	P13	P 13	Com.variety	IPGRB	Thessaloniki
21	P14	P 14	Com. variety	IPGRB	Thessaloniki
22	Bachovitiki	Bachovitiki	Cons. variety	Farmer’s collection	Pella
23	Ierapetras	Kafteri Ierapetras	Com. variety	IOSV	Thessaloniki
24	Platika Florinis	Platika Florinis	Com. variety	IPGRB	Thessaloniki
25	Florinis	Florinis	Com. variety	IPGRB	Thessaloniki

## Data Availability

Data available upon reasonable request.
